# Paris’ Pharmacologia

**Published:** 1844-03

**Authors:** 


### PARIS' PHARMACOLOGIA.

A new edition of this work, which has been so long one of the great favorites of the profession, has been published by the Harpers, of New York, in a single volume, having been re-written by the author to bring it up to the existing state of the science. Not a word in commendation of it is necessary, and we only notice it now to give information to our readers, that this new edition places the work within their reach. The present is a re-print of the ninth London edition. The fundamental doctrines of the work remain as at first announced, but, says the author, "so rapid and extraordinary has been the advancement of every branch of science during the last ten years, that its very language has become obsolete; a new Pharmacopœia had appeared during this interval, enriched with newly-discovered substances and novel preparations, and with a nomenclature radically changed. The science of chemistry had also, by the aid of quantitative analysis, assumed a new aspect; so that it was impossible, without remodelling the whole work, to render it consistent with the science of the day." By the performance of this necessary task, Dr. Paris has not only kept his work from becoming obsolete, but brought it before the public in a shape which will continue it for a long time in the hands of those who are studying "the theory and art of prescribing."

The work is for sale at the bookstore of Messrs. Morton & Griswold, in this city. Y.

